# Effect of defibrillation on the performance of an implantable vagus nerve stimulation system

**DOI:** 10.1186/s42234-021-00064-w

**Published:** 2021-03-16

**Authors:** Imad Libbus, Scott R. Stubbs, Scott T. Mazar, Scott Mindrebo, Bruce H. KenKnight, Lorenzo A. DiCarlo

**Affiliations:** LivaNova USA, Inc, 100 Cyberonics Blvd., TX 77058 Houston, USA

**Keywords:** Autonomic regulation therapy, Vagus nerve stimulation, Heart failure, Implantable cardioverter‐defibrillator, Defibrillation

## Abstract

**Background:**

Vagus Nerve Stimulation (VNS) delivers Autonomic Regulation Therapy (ART) for heart failure (HF), and has been associated with improvement in cardiac function and heart failure symptoms. VNS is delivered using an implantable pulse generator (IPG) and lead with electrodes placed around the cervical vagus nerve. Because HF patients may receive concomitant cardiac defibrillation therapy, testing was conducted to determine the effect of defibrillation (DF) on the VNS system.

**Methods:**

DF testing was conducted on three ART IPGs (LivaNova USA, Inc.) according to international standard ISO14708-1, which evaluated whether DF had any permanent effects on the system. Each IPG was connected to a defibrillation pulse generator and subjected to a series of high-energy pulses.

**Results:**

The specified series of pulses were successfully delivered to each of the three devices. All three IPGs passed factory electrical tests, and interrogation confirmed that software and data were unchanged from the pre-programmed values. No shifts in parameters or failures were observed.

**Conclusions:**

Implantable VNS systems were tested for immunity to defibrillation, and were found to be unaffected by a series of high-energy defibrillation pulses. These results suggest that this VNS system can be used safely and continue to function after patients have been defibrillated.

## Introduction

Heart failure (HF) is characterized by hemodynamic abnormalities that result in, and are exacerbated by, a marked imbalance created by increased sympathetic activity and withdrawal of parasympathetic tone. This pronounced pathological adrenergic hyperactivation contributes to the progression of HF, and increases the risk of mortality and morbidity independent of left ventricular ejection fraction (EF) and ventricular arrhythmias [1].

**Fig. 1:**
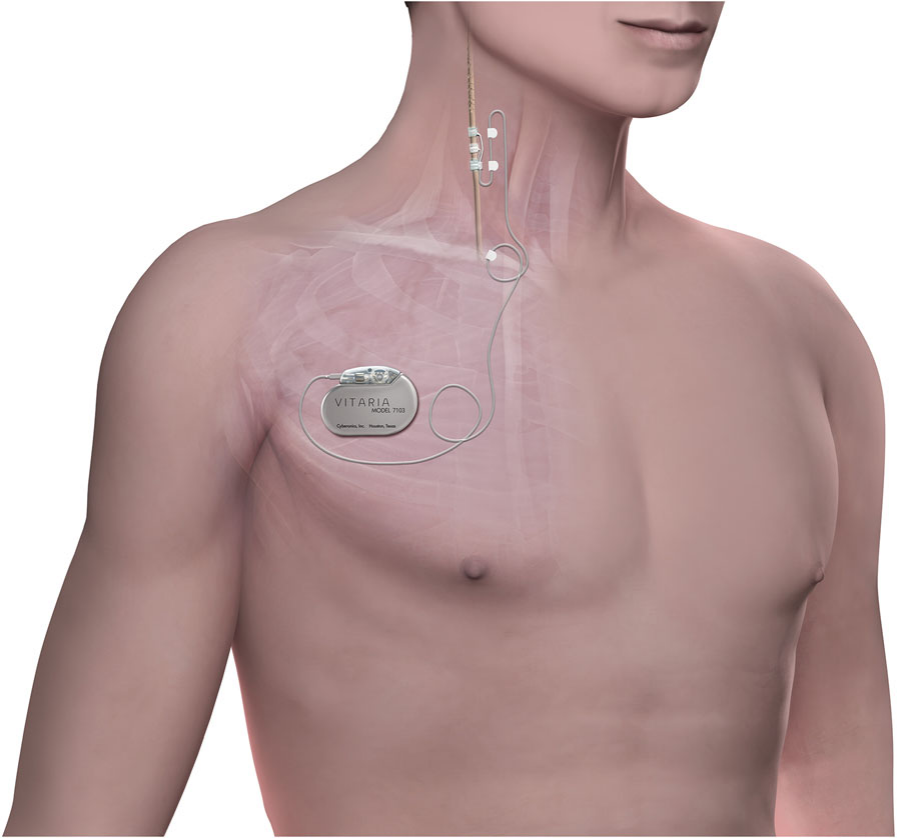
The VITARIA Autonomic Regulation Therapy (ART) system implanted on the right cervical vagus nerve, which consists of a Pulse Generator and lead

Autonomic Regulation Therapy (ART) is a novel investigational approach for the management of HF. ART utilizes cervical vagus nerve stimulation (VNS) to increase parasympathetic activity and to restore autonomic balance. ART is delivered using chronic stimulation through a self-sizing lead that is placed on the cervical vagus nerve without requiring any mapping for placement (Fig. 1). ART using open-loop VNS has been shown in a pilot study to be associated with long-term improvement in left ventricular function, 6-minute walk distance, NYHA class, heart rate, heart rate variability, and quality of life in patients with HF and reduced EF (HFrEF), [2–4] and is being evaluated further in an ongoing mortality and morbidity pivotal study in patients with HF and reduced left ventricular ejection fraction [5].

International guidelines recommend implantable cardioverter defibrillator (ICD) therapy for patients with HFrEF and at risk for ventricular arrhythmias [6–9]. Because HFrEF patients with implanted ART may be exposed to either external or internal high-energy defibrillation, it is important to evaluate whether defibrillation energy has an effect on the operation of the ART system. The purpose of this study was to determine the degree to which an ART system may be affected by an applied defibrillation shock.

## Methods

International standards establish requirements for medical devices, and specify the parameters for defibrillation testing for active implantable devices. Testing was performed according to the protocol specified in ISO 14708-1, Implants for Surgery – Active Implantable Medical Devices – Part 1: General Requirements for Safety, Marking and for Information to be Provided by the Manufacturer [10].

**Fig. 2:**
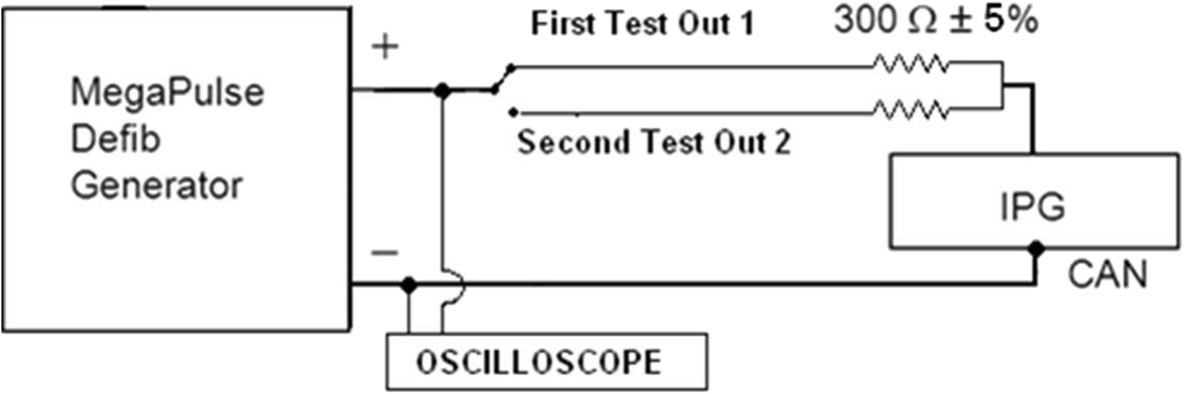
Schematic diagram of defibrillation test setup

Three Implantable Pulse Generators (IPG; Model 103, LivaNova, Houston, Texas) were tested using a defibrillation test voltage generator (MegaPulseDefib and MegaPulse Biphasic P, Compliance West, San Diego, CA). All equipment was calibrated and certified prior to performing the test. The outputs of the test voltage generator were sequentially connected to each of the two bipolar outputs of the IPG through a 300 Ω resistor and to the titanium case (Fig. 2).

**Fig. 3:**
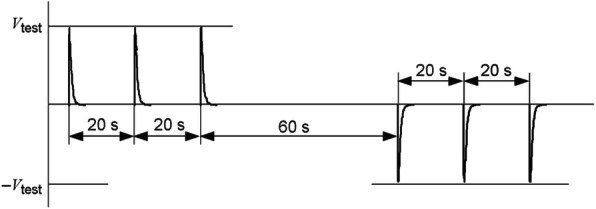
Timing of applied voltage pulses

A series of voltage spikes, monophasic pulses, and biphasic pulses were applied; three pulses with normal polarity 20 seconds apart, followed by a 60 second pause, followed by three pulses with reverse polarity 20 seconds apart (Fig. 3). The magnitude of the applied test pulses was 140 V, as specified by the international standard. Each IPG was exposed to three sets of pulses:

1. A damped sinus defibrillation waveform with rise time of 1.5–2.5 ms, with a pulse shape specified by ISO 14708-1 clause 20.2.
2. A monophasic, truncated exponential waveform with 10 ms duration.
3. A biphasic, truncated exponential waveform with 10 ms duration.

According to ISO 14708-1 clause 20.2, there should be no irreversible damage to the IPG after completion of the test. Each IPG was interrogated before and after each test, and a comparison of programmed device parameters was performed. After all tests were completed, each IPG underwent a final electrical test, a duplicate of what is performed at the end of the manufacturing process to confirm that the device met all functional specifications. Programmed values (output current, output pulse width, output pulse rise time, output pulse fall time, output pulse overshoot, pulse rate accuracy, magnet response, device reset, battery voltage, and others) were programmed and measured to confirm that they produce an output that is within the allowable tolerance.

## Results

All defibrillation tests were performed successfully in all devices, with no deviations from the ISO 14708-1 mandated protocol. The interrogated data from all three IPGs after each test was unchanged from the programmed values prior to the test. All three devices passed the post-test final electrical test, with no failures or parameter shifts.

## Discussion

Patients with HF and reduced EF are at risk for life-threatening ventricular arrhythmias, and these patients commonly receive ICD therapy and external DF. These patients are also being evaluated for ART, a novel therapy that uses a VNS IPG to modulate autonomic balance. This raises the question of whether an implantable ART system is able to survive the application of DF energy and continue to deliver therapy as originally programmed. This study sought to test the ART system according to ISO 14708-1, the international standard for DF immunity for implantable medical devices.

All three devices met the requirements established by the international standard for DF immunity testing. Following the application of three different sets of DF test pulses (different waveforms, including opposite polarity pulses), the settings of all tested devices were unchanged, and the devices passed the final electrical test, indicating that all functions were within manufacturer specifications.

Although this DF immunity test was designed to simulate the conditions that an implantable device would experience in the presence of DF energy, it is still only a benchtop test. Future research is needed to complement this test by determining whether this ART system is able to withstand energy from internal and external DF *in vivo*.

## Conclusions

Implantable VNS systems equivalent to the VITARIA ART system were tested for immunity to DF, and were found to be unaffected by a series of high-energy DF pulses. The results of this study suggest that the ART system can be used safely and continue to function after patients have been defibrillated.

## Data Availability

The datasets from the current study are available from the corresponding author on reasonable request.
